# Reconstructing the First Metatarsophalangeal Joint of *Homo naledi*

**DOI:** 10.3389/fbioe.2019.00167

**Published:** 2019-07-10

**Authors:** Yuxuan Fan, Djorđje Antonijević, Svetlana Antic, Ruining Li, Yaming Liu, Zhiyu Li, Marija Djuric, Yifang Fan

**Affiliations:** ^1^Foot Research Laboratory, School of Physical Education and Sport Science, Fujian Normal University, Fuzhou, China; ^2^Laboratory for Atomics Physics, Institute for Nuclear Sciences “Vinca”, University of Belgrade, Belgrade, Serbia; ^3^Laboratory for Anthropology, School of Medicine, Institute of Anatomy, University of Belgrade, Belgrade, Serbia; ^4^Center for Radiological Diagnostics, School of Dental Medicine, University of Belgrade, Belgrade, Serbia; ^5^College of Foreign Studies, Jinan University, Guangzhou, China

**Keywords:** reconstruction, body coordinate system, first metatarsophalangeal joint, fossil, sesamoid groove

## Abstract

The aim of the present study was to develop a new method to reconstruct damaged metatarsophalangeal joint (MTPJ) of *Homo naledi*'s fossil and to deepen the understanding of the first metatarsal head (FMH) morphological adaptation in different gait patterns. To this purpose three methods were introduced. The first served to compare the anthropometric linear and volumetric measurements of *Homo naledi*'s MTPJ to that of 10 various athletes. The second was employed to measure curvature diameter in FMH's medial and lateral grooves for sesamoid bones. The third was used to determine the parallelism between medial and lateral FMH grooves. The anthropometric measurements of middle-distance runner to the greatest extent mimicked that of *Homo naledi*. Thus, it was used to successfully reconstruct the damaged *Homo naledi*'s MTPJ. The highest curvature diameter of medial FMH groove was found in *Homo naledi*, while in lateral FMH groove it was the highest in volleyball player, suggesting their increased bear loading. The parallelism of medial and lateral FMH grooves was observed only in *Homo naledi*, while in investigated athletes it was dis-parallel. Athletes' dis-paralleled structures make first MTPJ simple flexion movement a complicated one: not rotating about one axis, but about many, which may result in bringing a negative effect on running. In conclusion, the presented method for the reconstruction of the damaged foot bone paves the way for morphological and structural analysis of modern population and fossil hominins' gait pattern.

## Introduction

In the last decade, running related injuries in sports have been investigated with considerable attention given to the differences in the running gait biomechanics, especially between habitually barefoot and shod runners, but also among various kinds of sport with their unique requirements (Murphy et al., 2013). Actually, barefoot runners tend to adopt, in most cases, the forefoot strike pattern (the runner lands the metatarsal area first and then continues with heel's contact on the ground) in contrast to the shod runners who use the rearfoot strike pattern (the runner lands heel first and then places metatarsal area down) (Jacob, 2001; Lieberman et al., 2010; Murphy et al., 2013). However, in professional shod running athletes, long term specific trainings might additionally influence the running gait pattern (Fourchet et al., 2015). The literature document numerous advantages of barefoot over shod gait pattern: three times lower average loading rate, lower angle and leg stiffness, higher energy efficiency during ground collision, and minimal impact transition of ground reaction force (Murphy et al., 2013; Reimann et al., 2018). Since it is reported that sport injuries, such as patellofemoral pain syndrome and tibial stress fractures occur with lower risk in barefoot runners, the shift to barefoot running pattern is suggested (Lohman III et al., 2011; Murphy et al., 2013). Additionally, we have to keep in mind that running pattern differs in todays' sports, which are associated with a different amount of risk to sports related injuries.

One of the crucial elements in running gait biomechanics is the first metatarsophalangeal joint (MTPJ), commonly known as “the big toe joint” (Saeki et al., 2015). This joint also articulates with two sesamoid bones on the plantar surface of the foot, and absorbs more than 50% of the forces acting in the forefoot during the gait (Dayton, 2018). Generating a high forward force (Goldmann et al., 2011), it influences not only motor function, but also the mechanical characteristics and lower extremity joints' compensation (Harton et al., 2002; Erdemir et al., 2004; Laroche et al., 2005). Any decrease in the stability of MTPJ certainly will impact the foot biomechanical characteristics either by reducing the motion range (Phillips et al., 1996), or by changing the regional plantar pressure with increasing the stress around the joint (Jacob, 2001; Kirane et al., 2008; Zhang et al., 2018). All these might lead to the joint and muscle overload, usually followed by common running related injuries: pattelofemoral pain syndrome, tibial stress fractures, plantar fasciitis and Achilles tendonitis (Murphy et al., 2013).

Knowing the fact that, prior to invention of the primitive shoes, humans were running barefoot, we supposed that MTPJ had to pass through a morpho-functional and structural adaptation. As we assumed that there are structural differences in MTPJ between the barefoot ancestors and todays' shod runners it was of interest to compare them, in order to give an answer if the barefoot running really has the advantage in the aspect of possible injury prevention. This might also light upon the structural and mechanical adaptations within foot arch to store and release elastic energy during barefoot vs. shod running pattern (Lucas et al., 2018). Since, in today's athletes, MTPJ might also pass through the additional adaptive morphology and structure to meet specific activity's requirement (Frost, 1997; Jacob, 2001), it was also of interest to compare the morphological characteristics of the MTPJ belonging to the barefoot ancestor, to those of the athletes engaged in different sports. To answer this task, we needed a relevant MTPJ of the habitually barefoot running ancestor. It is often very complicated to do a precise analysis because the skeletal remains are usually damaged (Kalvin et al., 1995). So, the aim of the present study, in the first line, was to develop a method to reconstruct the damaged fossil's first MTPJ. In the second line, the purpose was to clarify and deepen the understanding of the first metatarsal head (FMH) morphological adaptation in different gait patterns. We hypothesize that there are structural differences in MTPJ between the barefoot ancestors and today's shod runners.

## Subjects and Methods

### Subjects

This study was approved by the Ethics Committee of Fujian Normal University (No. FJNUSPE20170701). Since fundamental human movements include walking, running, jumping, object manipulation and so on (Chapman and Fraser, 2008), we choose typical sport events that involve basic movements: basketball—walking, running, jumping, throwing; badminton—object manipulation; volleyball—vertical jumping and whipping; wresting—pulling and pushing; triple jumping—sprint and jumping; middle-distance running—endurance running. Ten professional athletes (4 basketball players, 2 badminton players, 1 volleyball player, 1 wrestler, 1 triple jumper, and 1 middle-distance runner) were selected and their right feet were scanned by Computed Tomography (CT) (TOSHIBA/Aquilion ONE) in 0.5 mm thickness. The scanner was set to 120 kVp and 50 mA.

All participants had no injury, nor skeletal muscle disease, including 8 males (mean age: 25 ± 3 years; mean height: 171 ± 5 cm; mean weight: 69 ± 4 kg) and 2 females (mean age: 23 ± 0 years; mean height: 163 ± 2 cm; mean weight: 51 ± 1 kg). All participants provided fully informed consent to participate in the study by signing a written consent form. The test was conducted in accordance with the approved guidelines.

The fossil skeletons of *Homo naledi* (*H. naledi*) found in Dinaledi Chamber, South Africa in 2015, has a relatively complete first MTPJ (Berger et al., 2015). It belongs to endurance running hunter capable to pursue prey for long distances. With first metatarsal and its proximal and distal phalanges, the first MTPJ provides a valuable research object (Harcourt-Smith et al., 2015). Unfortunately, medial and lateral sesamoids of MTPJ are missing, while serious damage is found in joint head and fossa, and in distal phalange. In our previous investigations, a novel method is proposed to realign the sets of metatarsal bones by using their center of mass (COM) and principal axis of inertia (PAI) (Pavei et al., 2017; Arvin et al., 2018). It was successfully employed to reconstruct the human's metatarsal bones as well as erythrocytes from the capillary and human foot (Fan et al., 2017). In the present paper, we improved the method in order to reconstruct the damaged bone of the *H. naledi*, calculate the curvature diameter (CD) of the FMH's medial and lateral groove for sesamoids, and determine the parallelism between FMH's medial and lateral grooves.

### Method to Reconstruct the Damaged *H. naledi*'s MTPJ

The FMH structure of *H. naledi* is an independent variable and that of middle-distance runner is a dependent one because the former is unknown before reconstruction while the latter is a given structure. First, we developed a method to reconstruct the damaged *H. naledi*'s foot bones and MTPJ by using the middle-distance runner joint's three-dimensional (3D) model which matched the anthropometric parameters of *H. naledi* to the greatest extent. It was performed by using the runner's first metatarsal, the proximal end of the first metatarsal and the distal phalange. The following steps were conducted during the reconstruction:

– Export the CT scanning images to the medical image processing software system Mimics (Mimics Research 17.0 for X64; Materialize, Leuven, Belgium) to create the first MTPJ's 3D models.
– Use Euler principal axis (EPA) from the 3D MTPJ's model to generate the runner's foot bone body coordinate system.
– Standardize the foot bone length, width and height along the body coordinate axis.
– Compare 10 athletes' foot bone 3D models with that of *H. naledi*'s (bones width, length, height, volume, surface area and moments of inertia around x, y and z axes)—Secure one 3D model from the athletes that is the most identical to the one from *H. naledi*.
– Substitute the fossil's foot bone body coordinate with that of the athlete, and overlap these two 3D models.
– Use the athlete's foot bone 3D model boundary to remodel the missing part of the fossil and make the incomplete foot bone of the fossil a complete one.
– Use the proportion of missing foot bone before and after standardizing the length, width, and height to restore the original size of the complete foot bone fossil's 3D model and thus finish remodeling of the damaged *H. naledi*'s MTPJ.

### Method to Calculate the CD of the FMH's Medial and Lateral Groove for Sesamoids

To determine the FMH morphological and functional adaptation, the FMH grooves in contact to medial and lateral sesamoid bones were analyzed. Even with the part of the FMH missing from *H. naledi*'s foot bone, its complete cross section enables to employ *H. naledi*'s foot bone 3D model for the further analysis. To that purpose the following steps were performed:

– Define the long axis of the first MTPJ's EPA based on the foot bone body coordinate system created by EPA;
– In Mimics Software System, when the cross section rotates along the first MTPJ's EPA's long axis (spindle long axis), take the first MTPJ's largest cross section area as the rotating base level;
– When the base level is defined, rotate the foot bone 3D model clockwise and counterclockwise along the direction of the long axis. The rotation unit is set to 0.5°.
– After rotation, the contour of the first MTPJ changes in the cross section. The morphology of the FMH reveals the position of the medial and lateral grooves and its maximum CD can be measured.
– Take the section with maximal CD of FMH's groove as its coronal section, i.e., the diameter of this coronal section is the CD of the first FMH groove.

Schematic representation of the FMH grooves CD measuring is given in **Figure 2**.

### Method to Determine the Parallelism Between FMH's Medial and Lateral Grooves

The FMH grooves are circumferential surfaces. We assume that these two circumferential surfaces were part of the cylindric surface (Lockwood et al., 2002; Mahaisavariya et al., 2002). The following steps were conducted in order to examine whether the medial and lateral FMH grooves are parallel or dis-parallel:

– The first metatarsal's body coordinate system is developed by its EPA. Take the first metatarsal's centroid as the origin of the body coordinate and thus standardize the first metatarsal coordinate system.
– Identify the largest groove of the first metatarsal along its long axis.
– Rotate the largest surface about the vertical axis of the first metatarsal coordinate system, draw a circle of the curvature of the lateral groove's cross section.
– When the CD is the smallest, it is the CD of the lateral FMH groove. In the same way, calculate the CD of the medial FMH groove. In the same cross section, when both medial and lateral grooves reach smallest CD, they parallel; otherwise, they do not. In the same way, when two cylindric sections parallel, two sections derived from the cylinder axis are two circles where the CD is the smallest. When one section of two cylindric surfaces is a circle while the other is an ellipse, it means they are dis-parallel.

## Results

The outcomes of 10 athletes' first MTPJ basic anthropometric parameters are shown in Table 1. The first MTPJ of *H. naledi* is relatively smaller than those of the athletes on average, but *H. naledi*'s first metatarsal bone volume distribution is more proportional. Namely, *H. naledi*'s proximal phalanx's volume is 13,292.19 mm^3^ and distal phalanx is 7,252.54 mm^3^. The ratio between them is less than twice while that of athletes is nearly three times or more. Its proximal phalange looks identical to that of athletes while its distal phalange is rounded and stronger than that of examined athletes.

**Table 1:** Linear and volumetric measurement of the first metatarsal joint bones in investigated subjects.

**Subjects**	**First metatarsal**					**Proximal phalanx**					**Distal phalanx**				
	**Length (mm)**	**Width (mm)**	**Height (mm)**	**Surface area (mm^**2**^)**	**Volume (mm^**3**^)**	**Length (mm)**	**Width (mm)**	**Height (mm)**	**Surface area (mm^**2**^)**	**Volume (mm^**3**^)**	**Length (mm)**	**Width (mm)**	**Height (mm)**	**Surface area (mm^**2**^)**	**Volume (mm^**3**^)**
Basketball player 1	100.00	43.54	32.99	9,684.50	52,171.00	50.46	31.07	41.20	4,193.20	17,466.58	36.61	22.06	38.00	2,524.30	8,159.31
Basketball player 2	100.00	43.23	33.11	9,731.10	52,165.56	49.27	34.15	42.61	4,176.39	17,202.76	33.53	28.01	39.04	2,484.64	7,839.71
Basketball player 3	100.00	51.33	39.16	11,547.93	70,468.14	50.63	47.72	46.18	5,142.94	22,534.32	28.34	24.64	35.60	2,433.78	8,523.04
Basketball player 4	100.00	51.30	39.34	11,488.95	69,268.92	49.11	49.71	44.19	5,088.57	22,108.22	27.36	26.43	35.09	2,398.16	8,262.04
Badminton player 1	100.00	48.92	39.23	11,617.66	73,287.57	53.38	38.52	44.71	5,154.73	23,424.27	32.04	32.90	40.73	2,904.84	9,696.46
Badminton player 2	100.00	48.92	38.45	11,622.25	73,069.31	53.67	32.66	42.34	5,133.38	23,296.07	35.90	27.60	40.06	2,930.24	9,810.71
Jumper	100.00	45.64	33.00	9,670.03	53,595.01	47.38	40.55	36.52	4,156.07	16,586.19	31.84	30.14	36.14	2,405.28	7,387.71
Wrestler	100.00	48.19	40.12	10,989.32	65,759.84	51.35	42.10	45.46	4,832.74	20,941.93	36.90	27.01	37.63	2,861.60	10,008.66
Volleyball	100.00	44.97	35.68	9,767.82	53,604.66	48.38	43.41	42.91	4,100.92	15,833.66	22.06	32.50	29.53	1,958.38	5,789.57
Runner	100.00	43.29	32.72	9,497.87	52,340.88	50.78	38.72	40.50	4,164.21	16,562.12	33.33	19.42	34.64	2,150.35	6,485.48
*H. naledi*	100.00	39.99	29.20	8,332.98	40,784.73	48.20	35.27	38.41	3,633.81	13,292.19	42.45	38.20	42.17	2,247.04	7,252.54

**Figure 1:**
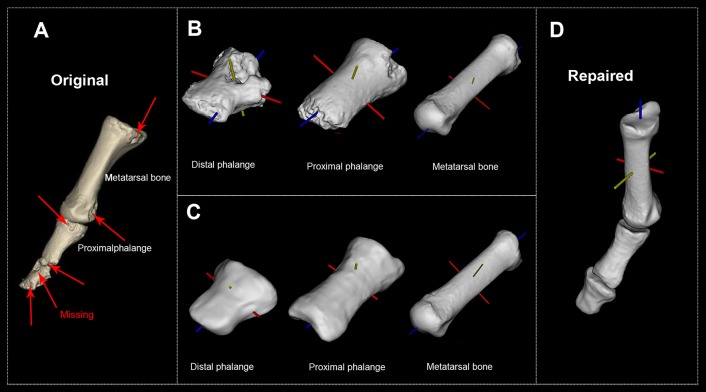
*H. naledi*'s first metatarsophalangeal joint before and after the reconstruction. **(A)** Original first MTPJ (Courtesy of the University of the Witwatersrand and the Dinaledi project provided access to these data originally appearing in, the collection of which was funded by. The files were downloaded from www.MorphoSource.org, Duke University). **(B)** Original distal phalange, proximal phalange, and metatarsal bone with their body coordinate system. **(C)** Reconstructed distal phalange, proximal phalange, and metatarsal bone with their body coordinate system. **(D)** Repaired and reconstructed first MTPJ with body coordinate system (see Supplementary Figure 1 for all 10 participants' reconstructed first MTPJ).

Every measurement value of athletes in Table 1 is normalized, and the ratio relation with *H. naledi* is obtained in Table 2. The statistical results show that the ratio of middle-distance runner (with the smallest mean value) is the closest to that of *H. naledi*. And the morphological observation of the middle-distance runner is also very close to that of *H. naledi*. *H. naledi*'s first MTPJ was repaired and reconstructed with reference to that of the middle-distance runner (see Figure 1).

**Table 2:** Comparison of linear and volumetric measurement of the first metatarsal joint bones in investigated subjects.

**Subjects**	**First metatarsal**					**Proximal phalanx**					**Distal phalanx**				
	**Length**	**Width**	**Height**	**Surface area**	**Volume**	**Length**	**Width**	**Height**	**Surface area**	**Volume**	**Length**	**Width**	**Height**	**Surface area**	**Volume**
Basketball player 1	1.0000	1.0888	1.1298	1.1622	1.2792	1.0469	0.8809	1.0726	1.1539	1.3140	0.8624	0.5775	0.9011	1.1234	1.1250
Basketball player 2	1.0000	1.0810	1.1339	1.1678	1.2790	1.0222	0.9682	1.1093	1.1493	1.2942	0.7899	0.7332	0.9258	1.1057	1.0810
Basketball player 3	1.0000	1.2836	1.3411	1.3858	1.7278	1.0504	1.3530	1.2023	1.4153	1.6953	0.6676	0.6450	0.8442	1.0831	1.1752
Basketball player 4	1.0000	1.2828	1.3473	1.3787	1.6984	1.0189	1.4094	1.1505	1.4003	1.6632	0.6445	0.6919	0.8321	1.0673	1.1392
Badminton player 1	1.0000	1.2233	1.3435	1.3942	1.7969	1.1075	1.0921	1.1640	1.4185	1.7623	0.7548	0.8613	0.9659	1.2927	1.3370
Badminton player 2	1.0000	1.2233	1.3168	1.3947	1.7916	1.1135	0.9260	1.1023	1.4127	1.7526	0.8457	0.7225	0.9500	1.3040	1.3527
Jumper	1.0000	1.1413	1.1301	1.1605	1.3141	0.9830	1.1497	0.9508	1.1437	1.2478	0.7501	0.7890	0.8570	1.0704	1.0186
Wrestler	1.0000	1.2051	1.3740	1.3188	1.6124	1.0654	1.1936	1.1835	1.3299	1.5755	0.8693	0.7071	0.8923	1.2735	1.3800
Volleyball	1.0000	1.1245	1.2219	1.1722	1.3143	1.0037	1.2308	1.1172	1.1285	1.1912	0.5197	0.8508	0.7003	0.8715	0.7983
Runner	1.0000	1.0825	1.1205	1.1398	1.2833	1.0535	1.0978	1.0544	1.1460	1.2460	0.7852	0.5084	0.8214	0.9570	0.8942
*H. naledi*	1.0000	1.0000	1.0000	1.0000	1.0000	1.0000	1.0000	1.0000	1.0000	1.0000	1.0000	1.0000	1.0000	1.0000	1.0000

Figure 1A is *H. naledi*'s original MTPJ (Harcourt-Smith et al., 2015). Figure 1B reveals that the damaged proximal phalange's head diminishes the accuracy of reconstruction. So, we repaired the distal phalange, proximal phalange and metatarsal bone (see Figure 1C). Figure 1D presents the repaired and reconstructed MTPJ. Arrows point to the missing/damaged zone while trusses are the EPAs.

**Figure 2:**
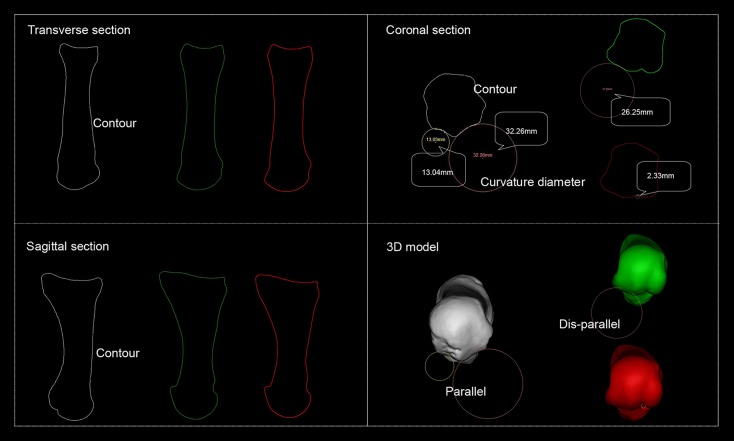
Schematic representation of the first metatarsal head's groove curvature diameter. White refers to *H. naledi*'s first metatarsal, green refers to the volleyball player first metatarsal's lateral groove reaches smallest CD and red to that of medial groove reaches smallest CD. Transverse, coronal, and sagittal section are defined by Mimics software system. On coronal section, *H. naledi*'s medial and lateral grooves of the first metatarsal reach smallest CDs while those from the volleyball player do not.

The FMH's medial and lateral grooves for sesamoids are fundamental structure of MTPJ. Their CD can reflect their function because form follows function. Using our method, we obtained *H. naledi*'s CDs of its grooves (see Figure 2).

Figure 2 shows transverse section, sagittal section, coronal section, and 3D model presented by Mimics software system. *H. naledi* FMH's medial and lateral groove CDs are on the same section, indicating that they parallel.

Notably, in badminton player, triple jumper, and basketball players, the sesamoid bones responsible for shaping the FMH grooves were absent or damaged and thus not suitable for CD calculations and parallelism analysis. Therefore, our further analysis was performed only in wrestler, volleyball player and middle-distance runner.

**Figure 3:**
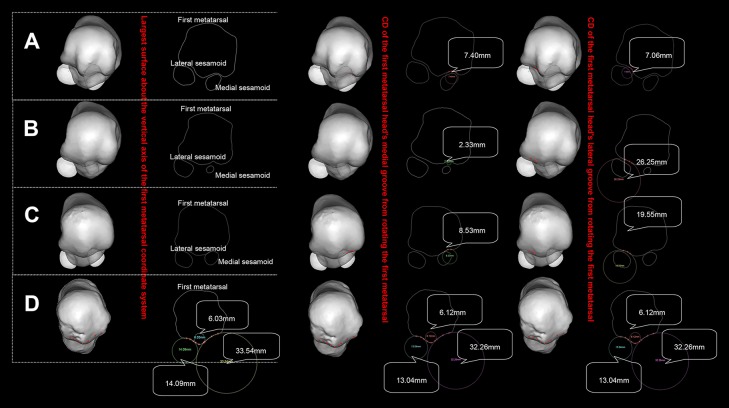
Posture of the standardized first metatarsal head. **(A)** refers to wrestler, **(B)** to volleyball player, **(C)** to middle-distance runners, **(D)** to *H. naledi* (see Supplementary Video 1 for an animation).

By rotating around the centroid, CDs of the FMH grooves for sesamoids are obtained (see Table 3). It shows that we only need rotate *H. naledi* with −2°, CDs of medial and lateral grooves are obtained, indicating that they parallel while those from wrestler, volleyball player and middle-distance runner do not. See Figure 3 for the posture of the standardized first MTPJ.

**Table 3:** Curvature diameters of the first metatarsal heads grooves for sesamoids and rotation angles of the reconstructed models around the centroid.

**Subjects**	**Medial**		**Lateral**	
	**Rotation angle (**°**)**	**Curvature diameter (mm)**	**Rotation angle (**°**)**	**Curvature diameter (mm)**
Wrestler	−1	7.40	−3	7.06
Volleyball player	−3	2.33	−2	26.25
Runner	−1	8.53	1	19.55
*H. naledi*	−2	32.26	−2	13.04

Table 3 and Figure 3 show that CDs from volleyball player and middle-distance runner FMH's medial grooves are far smaller than those from the lateral grooves while CDs of the wrestler are identical from both grooves. *H. naledi*'s CD of the medial groove is far greater than that of the lateral groove. Figure 3 shows that the groove's CD has no definite relation with the size of the sesamoid. Wrestler's medial sesamoid is greater than the lateral one while the CD of the lateral groove is smaller than that of the medial groove.

## Discussion

In this study, a novel method to fully reconstruct damaged MTPJ of *H. naledi* is presented. Proposed method consists of linear and volumetric comparison of the first metatarsal bone and phalanges in *H. naledi* to that of athletes. In brief, results of this study show that the *H. naledi*'s first MTPJ mimics that of the middle-distance runner. Thus, the middle-distance runner's first MTPJ is used as a reference to repair the incomplete *H. naledi*'s first MTPJ, to restore its original posture and to further enable the functional analysis of the investigated first MTPJ.

Presented method is useful to develop a deeper understanding of the morphological adaptation of FMH in various gait patterns. CDs of FMH groove differ among the investigated subjects, suggesting the correlation between their movement patterns on one side and the structure and morphology of MTPJ on the other side. Differences in metatarsal head morphology are presumably the result of different joint loading conditions (Sylos-Labini et al., 2017). The forces acting on the forefoot during the gait are distributed mainly along the first ray having the FMH bone absorbing 29% and big toe absorbing 23.8% of the body weight (Jacob, 2001). A fundamental study by Jacob demonstrated that a sum vector of all forces directed from the ground to the FMH is dependent on the following forces: ground reaction forces under the toe pad and metatarsal head and forces acting along the tendons of the flexor hallucis longus and flexor hallucis brevis in order to balance the force produced under the toe pad (Jacob, 2001). For those athletes who are subjected to vertical stress (volleyball player), their FMH groove CDs tend to be smaller while for those athletes who are subject to shear stress (runners), they tend to be greater. Medial CD in *H. naledi* is nearly four times higher than that in middle distance runner which can be explained by its barefoot induced forefoot strike gait pattern. In this pattern, the runner makes initial contact with the metatarsal area and then continue with a heel resulting in higher stress in metatarsal head than in rearfoot shod running where the runner lands heel first and then places the metatarsal bones down (Murphy et al., 2013). Yet, when comparing *H. naledi* to middle distance runner, it is noteworthy that in *H. naledi* CD of medial groove is two times higher than that of the lateral groove; the opposite holds true for middle distance runner having two times higher value of CD in the lateral than in the medial groove. Having in mind that the CD grossly corresponds with the stress absorbed through the sesamoid bones, obtained result may further help to highlight numerous unknown differences between barefoot (*H. naledi*) and rearfoot (middle distance runner) gait pattern. Namely, observed CDs suggest that in barefoot running, which is associated with forefoot strike pattern, higher stress is concentrated in the medial part of the foot, while in shod running, with rearfoot strike pattern it is located more laterally (Meyer et al., 2018). This implies endurance running hypothesis (Bramble and Lieberman, 2004) might be correct.

Wrestler put efforts to maintain balance and posture in all directions (anterior/posterior, lateral/medial) and hence the CDs are similar in lateral and medial groove. His lateral sesamoid extends outward while the tibial sesamoid flexes inward while the FMH groove is small and limits the sesamoid. In volleyball player, high value of FMH lateral CD corresponds with the large support area, which is necessary for the increased force bearing when suddenly jumping vertically (Stefanyshyn and Nigg, 1998). In the middle-distance runner, sesamoid ridge lies in the middle of the metatarsal head, with the medial and lateral sesamoids getting closer to the sesamoid ridge. Both sesamoid groove's CDs are relatively high. This is because running is mostly a forward movement and the first MTPJ plays an important role when stretching forward (Zifchock et al., 2019). Thus, the middle-distance runner needs to maintain the first MTPJ to exert force continuously and steadily (Oleson et al., 2005). The high CD of the medial and lateral grooves found in *H. naledi* facilitates the first MTPJ to bear force continuously and stably, which means that *H. naledi* has the ability to bear great loading bare-footedly. The first metatarsal of *H. naledi* is thinner, with its proximal phalange more like in modern humans, but its distal phalange is thicker and more massive than that of the athletes and therefore the first interphalangeal joint is considered agile. Compared with the relatively smaller skeleton, it represents *H. naledi*'s superior climbing ability over modern athletes. In addition, the massive distal phalange suggests that if MTPJ performs stretching movement, it has larger support area and subsequently it reduces the pressure peak values and increases the ability to grip the ground (Harcourt-Smith et al., 2015).

The results further show that *H. naledi*'s FMH lateral groove and medial groove parallel while those of the athletes do not. Figure 2 shows that only *H. naledi*'s medial and lateral grooves reach smallest CD in the same cross section, indicating that only *H. naledi*'s medial and lateral grooves parallel. Therefore, the rotations of medial and lateral grooves for sesamoid are equal while in athletes they differ. The significant differences in the angles observed for different athletes are presumably caused by the variations in the movement they conduct. Precisely, the first MTPJ does rotate around the coronal axis, but it also generates motion about the sagittal and vertical axis. Flexor hallucis brevis is attached to cuboid bone and phalanx proximalis, across the grooves of first metatarsal. Therefore, the parallelism of grooves could affect the attached point of it. When the medial and lateral grooves do not parallel, eccentric force increases; otherwise, eccentric force declines. The paralleled FMH grooves of *H. naledi* took form in barefoot condition. This parallelism of grooves boosted the function of flexor hallucis brevis. However, the disappearance of this parallelism in modern human being's foot could be attributed to wearing shoes, which implies wearing shoes might impact the structure of our FMH. Such relations can also help answer some still unclear questions about the risk for the sports injuries in modern athletes. Dis-paralleled structure turns athletes' first MTPJ simple flexion movement into a complicated one: not rotating around one axis, but around many, which brings negative effect on running. Shod running might be the factor leading to this structural change. Further systematic investigations on barefoot vs. shod running caused alterations in FMH may possibly help to prevent plantar fasciitis and other shod running related injuries.

The method introduced in the current study is relevant, considering that it clarifies some unknown issues in the human gait pattern modalities. The FMH groove selected for the analysis lies in the maximal cross section along long axis of the first metatarsal. Chosen cross section reveals the position of sesamoid when it bears load, cushions and absorbs shock. *H. naledi*'s more economical foot arch structure sheds light on the issue of the barefoot running, having in mind that shod running is considered to be the main cause of the plantar fasciitis in humans nowadays (Chen et al., 2019). *H. naledi* runs barefoot regularly, yet its MTPJ structure shows that its barefoot endurance running is more economical than in modern athletes while at the same time the risk for injury is lower than that in the athletes. This verifies our hypothesis that structural differences exist in MTPJ between the barefoot ancestors and today's shod runners. This point toward wearing simple shoes to maximize the function of MTPJ (Lieberman et al., 2010), frees the feet just like how the primates walked in Africa and restores the health to the natural best state.

In this study, the participants were all professional athletes, so only a limited number of participants from each sport event were recruited. We were unable to enroll the same number of participants for all sports in this study. Also, the selection of participants was not comprehensive enough. In our future research, we will recruit a balanced number of participants from different age, weight, height range and different population groups with a balanced number of male and female participants to obtain more accurate results.

## Conclusion

The novel method introduced in the present study was utilized to accurately restore and reconstruct *H. naledi*'s first MTPJ. The FMH grooves CDs of *H. naledi* were compared with those from the wrestlers, volleyball player and the middle-distance runner. The morphological pattern of middle-distance runner to the greatest extent matches that of the *H. naledi*, while in wrestlers and volleyball players MTPJ went through the considerable morphological and functional adaptation. The method may help to explain morphological adaptation in FMH depending on the activity performed. *H. naledi* barefoot gait pattern seems more protective for FMH since parallelism of its medial and lateral FMH grooves prevents undesirable rotations around sagittal and vertical axes. In contrast, proposed method suggests that dis-parallelism of FMH medial and lateral grooves may cause unwanted rotations in wrestler, volleyball player and middle-distance runner. Taking all observed lines of evidence into consideration, it can be postulated that the proposed method may be useful to clarify numerous still unanswered questions regarding the physiological function of the foot and the development of the foot injuries. In the future studies, the method presented here will be employed to compare the morphological and functional differences between endurance runners in modern and ancient populations, in the light of shod and barefoot running. This method could also be applied to other types of bones.

## Data Availability

3D surface data and other data of *Homo naledi* are available from https://www.morphosource.org/. According to Harcourt-Smith et al. (2015), the fossil was scanned using the Next Engine desktop scanner. Codes from the foot remains are: U.W. 101-1443 Metatarsal 1, U.W. 101-1551 Distal hallucial phalanx, and U.W. 101-1419 Proximal hallucial phalanx.

Ten professional athletes' data are available upon request to the corresponding author.

## Ethics Statement

This study was carried out in accordance with the recommendations of Professional Guidelines for Fujian Normal University, Fujian Normal University Teachers' Committee with written informed consent from all subjects. All subjects gave written informed consent in accordance with the Declaration of Helsinki. The protocol was approved by the Ethics Committee of Fujian Normal University.

## Author Contributions

YiF and MD conceived the study. YuF, RL, and YL collected and analyzed data. YiF, YuF, DA, SA, and ZL wrote the manuscript and all authors revised the final manuscript.

### Conflict of Interest Statement

The authors declare that the research was conducted in the absence of any commercial or financial relationships that could be construed as a potential conflict of interest.

## Supplementary Material

Supplementary Figure 1 Posture of the first MTPJ of 10 scanned athletes positioned with each bone's body coordinate system.

Supplementary Video 1 A circular view of 3D model of the first MTPJs of three athletes and *H. naledi* (green refers to the first MTPJ of wrestler, blue to volleyball player, red to middle-distance runner, and white to *H. naledi*).

## Abbreviations

*Homo naledi*: *H. naledi*
MTPJ: metatarsophalangeal joint
FMH: first metatarsal head
EPA: Euler principal axis
CD: curvature diameter.
