# Supplementary figures and images for: Reconstructing the First Metatarsophalangeal Joint of Homo naledi
Source: Front Bioeng Biotechnol. 2019 Jul 10;7:167. doi: 10.3389/fbioe.2019.00167 (PMC6635694; doi:10.3389/fbioe.2019.00167)

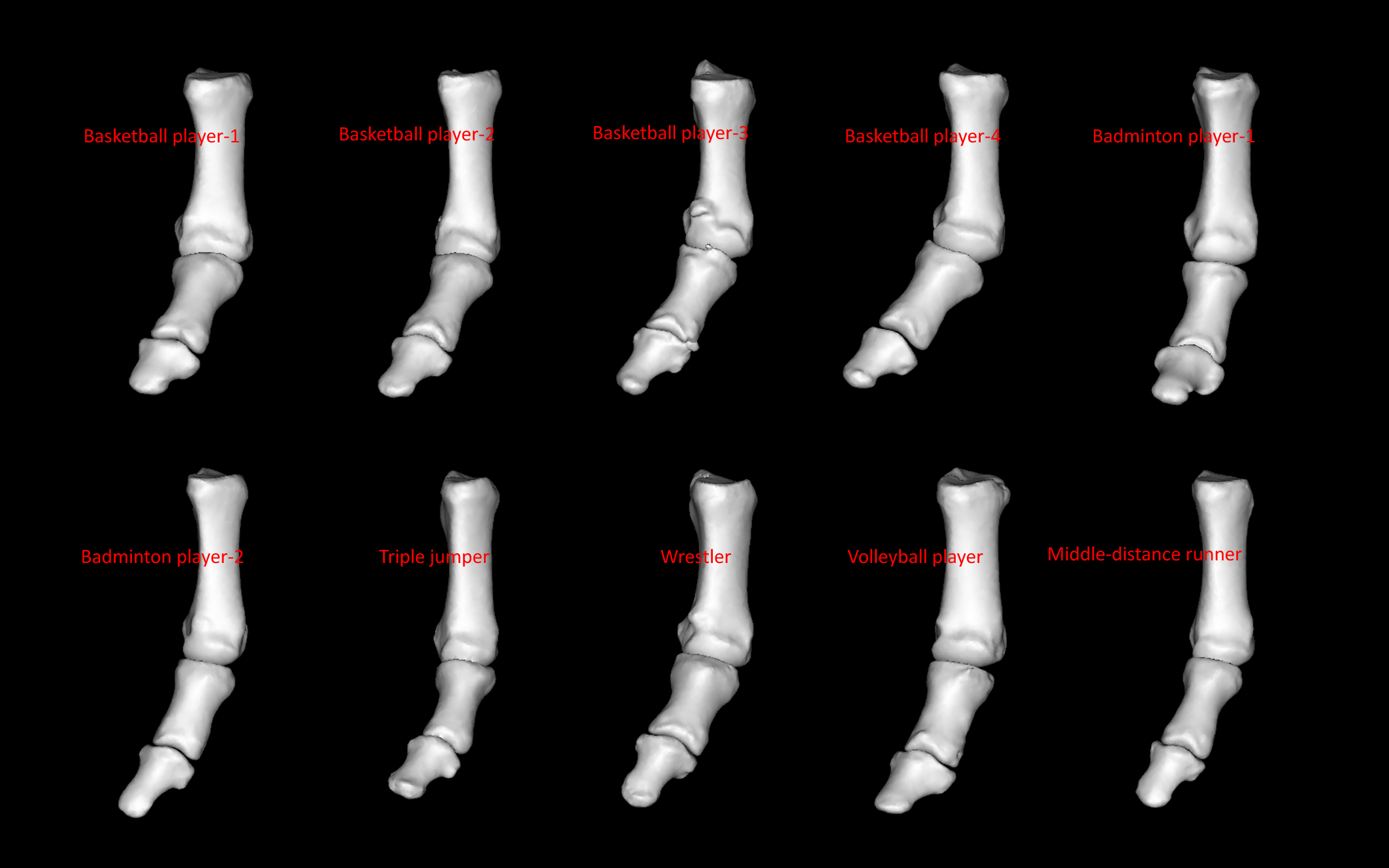

Supplement: Supplementary Figure 1 — Posture of the first MTPJ of 10 scanned athletes positioned with each bone's body coordinate system. [file Image_1.tif]
